# Supplementary material for: Prenatal Exome Sequencing in Recurrent Fetal Structural Anomalies: Systematic Review and Meta-Analysis
Source: J Clin Med. 2021 Oct 15;10(20):4739. doi: 10.3390/jcm10204739 (PMC8538791; doi:10.3390/jcm10204739)
Supplement: Supplementary file 1 [file jcm-10-04739-s001.zip › jcm-1359142-supplementary.pdf]

Supplementary 1. Search strategy and query syntaxes used in this systematic review.

Scoping review: "exome sequencing"[All Fields] AND "fetuses"[All Fields] AND "recurrent"[All Fields]

Iterative review:

Search: ((exome) AND (ultrasound) AND (prenatal diagnosis)) AND NOT (review)

("exome"[MeSH Terms] OR "exome"[All Fields] OR "exomes"[All Fields] OR "exomic"[All Fields]) AND ("diagnostic imaging"[MeSH Subheading] OR ("diagnostic"[All Fields] AND "imaging"[All Fields]) OR "diagnostic imaging"[All Fields] OR "ultrasound"[All Fields] OR "ultrasonography"[MeSH Terms] OR "ultrasonography"[All Fields] OR "ultrasonics"[MeSH Terms] OR "ultrasonics"[All Fields] OR "ultrasounds"[All Fields] OR "ultrasound s"[All Fields]) AND ("prenatal diagnosis"[MeSH Terms] OR ("prenatal"[All Fields] AND "diagnosis"[All Fields]) OR "prenatal diagnosis"[All Fields]) AND ("review"[Publication Type] OR "review literature as topic"[MeSH Terms] OR "review"[All Fields])

Translations

exome: "exome"[MeSH Terms] OR "exome"[All Fields] OR "exomes"[All Fields] OR "exomic"[All Fields]

ultrasound: "diagnostic imaging"[Subheading] OR ("diagnostic"[All Fields] AND "imaging"[All Fields]) OR "diagnostic imaging"[All Fields] OR "ultrasound"[All Fields] OR "ultrasonography"[MeSH Terms] OR "ultrasonography"[All Fields] OR "ultrasonics"[MeSH Terms] OR "ultrasonics"[All Fields] OR "ultrasounds"[All Fields] OR "ultrasound's"[All Fields]

prenatal diagnosis: "prenatal diagnosis"[MeSH Terms] OR ("prenatal"[All Fields] AND "diagnosis"[All Fields]) OR "prenatal diagnosis"[All Fields]

review: "review"[Publication Type] OR "review literature as topic"[MeSH Terms] OR "review"[All Fields]

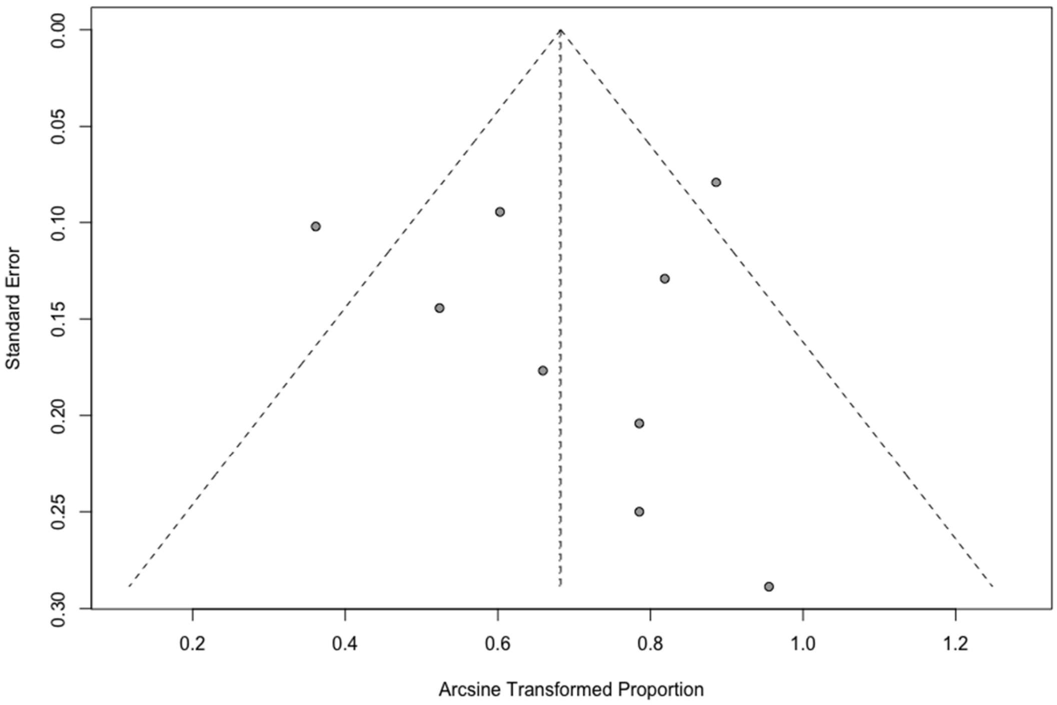

Figure S1. Funnel plot analysis for the asymmetry of publication among small studies.
